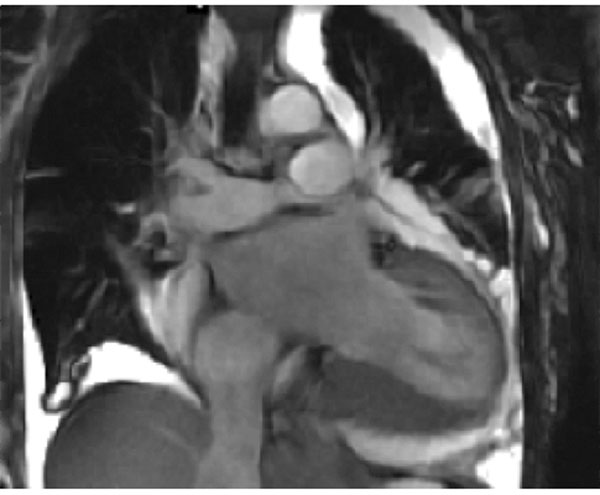# The early role of CMR in the assessment of cardiomyopathy

**DOI:** 10.1186/1532-429X-14-S1-P146

**Published:** 2012-02-01

**Authors:** Matthew Barrett, Deirdre F Waterhouse

**Affiliations:** 1 SVUH, Dublin, Ireland; 2 CMR Department, Blackrock Clinic, Dublin, Ireland

## Summary

We performed a one year study to assess the impact of CMR imaging on the management of patients with suspected or confirmed cardiomyopathy.

## Background

Investigation and risk stratification of suspected or confirmed cardiomyopathy traditionally involves correlation between electrocardiographic, echocardiographic and angiographic findings in an appropriate clinical setting. Cardiac Magnetic Resonance (CMR) is the new gold standard in assessment of cardiac structure, function and perfusion, provided in a single study. Aetiology and prognostic factors may be investigated concurrently.

## Methods

This was a single centre, 12-month experience of patients referred for assessment of presumptive cardiomyopathy. Images were provided by a 1.5T GE Scanner and were reported by a Level 3 Reporter.

## Results

224 patients (145 male, 79 female) underwent CMR assessment of cardiomyopathy. 177 (79%) were outpatient referrals.

The primary indications for CMR included

49 (21.9%) - Arrhythmia

42 (18.8%) - Abnormal echocardiogram with no cause found

35 (15.6%) - Cardiac symptoms with normal coronaries

32 (14.3%) - Screening for family history of CM/SCD

23 (10.3%) - Abnormal ECG/holter/stress test

18 (8%) - Follow-up of previously diagnosed CM

17 (7.6%) - Systemic illness

7 (3.1%) - ICD insertion

CMR provided sufficient information to confirm or outrule cardiomyopathy in 68.2% of cases. A new diagnosis of cardiomyopathy was made in 25.4%. CMR also had an important role in ongoing assessment of patients with established diagnosis of cardiomyopathy, with 22.2% having their previous diagnosis outruled and 27.8% being recommended for device implantation as a direct result of CMR findings.

Overall in cardiomyopathy assessment, CMR had an impact on management in 50% of patients, with a therapeutic consequence on 36.2%, including medication changes, angiography and device insertion.

## Conclusions

Patients at all stages of the clinical spectrum of cardiomyopathy, from initial presentation to institution of therapy and long-term follow up may benefit from CMR.

CMR should be implemented early in the diagnostis of suspected cardiomyopathy. Our data demonstrates the significant impact CMR consistently has on confirming diagnosis, guiding therapy and providing accurate prognosis in this patient group.

## Funding

None.

**Table 1:** Baseline Characteristics

Gender	Male - 145	Female - 79
Referral Source	OPD - 177	Inpatient - 47
BMI	Mean - 26.7 kg/m2	StDev - 4.4 kg/m2
Age	Mean - 63.4 years	StDev - 16.7 years

**Figure 1:**